# 

**Published:** 1890-05

**Authors:** 


### HALL'S JOURNAL OF HEALTH

ESTABLISHED 1854

CONTENTS.

VOL. 37. No. 5.

| | PAGE. |
|---|---|
| A Change for the Better | 97 |
| Amusements | 97 |
| Calisthenics | 100 |
| Are New Yorkers Losing their Eyesight | 103 |
| Cod Liver Oil | 105 |
| Eggs | 106 |
| Good Rules for Dyspeptics | 107 |
| How to Walk and Sit | 108 |
| The Canal of Joseph | 109 |
| Electric Currents of the Skin | 110 |
| Trephining for Insanity | 110 |
| Medicinal Uses of Coffee | 111 |
| Round Shoulders | 111 |
| Colic | 112 |
| Woman's Dress | 112 |
| A Mummy Unrolled | 113 |
| The Educational Value of Outdoor Games | 114 |
| Vegetarianism | 115 |
| The Goldsmith Piano | 115 |
| Hygiene of Carpets | 116 |
| The Fever Microbe | 116 |
| Household | 117 |
| Miscellaneous | 118 |
| Literary | 120 |
| May | 120 |

INDEX TO ADVERTISEMENTS OF HALF A PAGE AND OVER.

| | PAGE. |
|---|---|
| An Unprecedented Offer | I |
| Celluloid | II |
| Hollow Suppositories | III |
| Christ Before Pilate | IV |
| Hall's Journal of Health | V |
| Goldsmith's Pianos | V |
| E. C. Morris & Co | VI |
| Dennin's Certain Cure | VII |
| Revised Premium List | VIII |
| Hospital Remedy Co. | IX |
| Herb Cure Co. | X |

